# How and why do root apices sense light under the soil surface?

**DOI:** 10.3389/fpls.2015.00775

**Published:** 2015-09-24

**Authors:** Mei Mo, Ken Yokawa, Yinglang Wan, František Baluška

**Affiliations:** ^1^College of Biological Sciences and Biotechnology, Beijing Forestry University, Beijing, China; ^2^Institute of Cellular and Molecular Botany, University of Bonn, Bonn, Germany; ^3^Department of Biological Sciences, Tokyo Metropolitan University, Tokyo, Japan

**Keywords:** root, photomorphogenesis, photoreceptors, plant, phytohormones, phototropism, auxin

## Abstract

Light can penetrate several centimeters below the soil surface. Growth, development and behavior of plant roots are markedly affected by light despite their underground lifestyle. Early studies provided contrasting information on the spatial and temporal distribution of light-sensing cells in the apical region of root apex and discussed the physiological roles of plant hormones in root responses to light. Recent biological and microscopic advances have improved our understanding of the processes involved in the sensing and transduction of light signals, resulting in subsequent physiological and behavioral responses in growing root apices. Here, we review current knowledge of cellular distributions of photoreceptors and their signal transduction pathways in diverse root tissues and root apex zones. We are discussing also the roles of auxin transporters in roots exposed to light, as well as interactions of light signal perceptions with sensing of other environmental factors relevant to plant roots.

## Introduction

**FIGURE 1:**
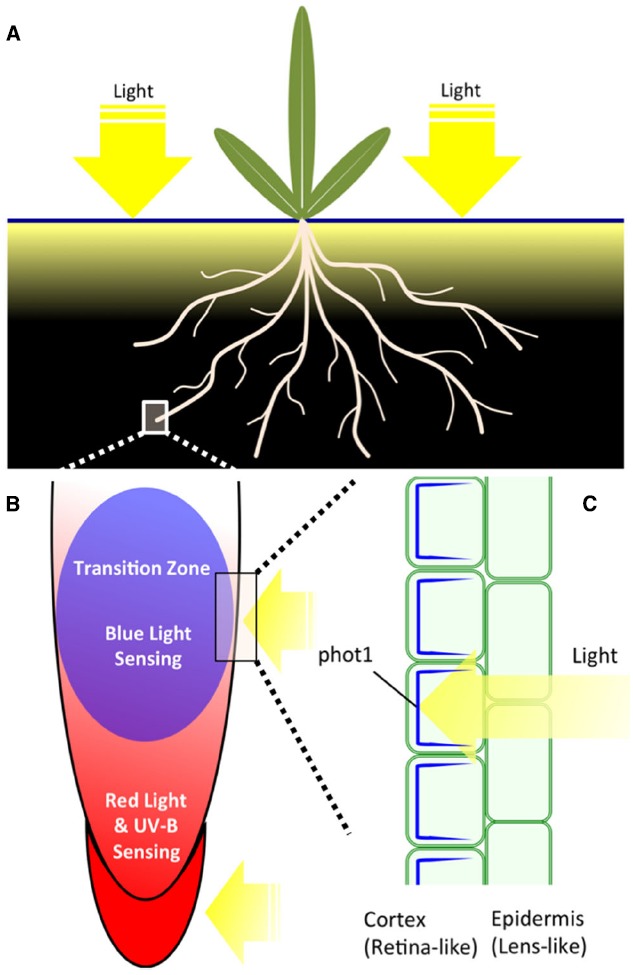
**(A)** Plant organs and their light environment. Shoot part of plants is fully exposed to light during a day. Root part is exposed only to limited amounts of light which penetrates into the soil during a day. Actual light mosaics in the soil depend on numerous factors and it changes with the depth (Woolley and Stoller, 1978; Tester and Morris, 1987; Kasperbauer and Hunt, 1988). **(B)** Root apex zonation with respect of light-sensitivity. Root cap, meristem and transition zone are expressing phytochromes (Adam et al., 1994; Somers and Quail, 1995a,b; Goosey et al., 1997) whereas only the transition zone is abundantly expressing phototropin phot1 (Wan et al., 2008, 2012). UVR8 is expressed, similarly as phytochromes, in all zones of *Arabidopsis* root apex (Rizzini et al., 2011; Yokawa et al., 2014). RUS1 and RUS2 are also expressed preferentially in cells of the transition zone (Leasure et al., 2009; Yu et al., 2013). **(C)** Tissue-specific and polar distribution of phot1 in cells of the transition zone. While epidermis cells do not express phot1, this blue light photoreceptor essential for negative phototropism of roots is abundant and polarly distributed (shown in blue) in underlying cortex cells (Wan et al., 2008) and controls PIN2 distribution and recycling (Wan et al., 2012). This tissue-specific expression and polarity of phot1 fits nicely to the plant “ocelli” concept (the epidermis act as lens-like tissue and the sub-epidermis as retina-like tissue) as proposed by Haberland for shoots (Haberlandt, 1904; Darwin, 1907; von Guttenberg, 1955).

Roots, the underground organ of all terrestrial plants, do not grow in a completely dark environment. Actually, sunlight can penetrate several millimeters beneath the soil surface, affecting the development of root architecture and guiding the growth direction of roots (Woolley and Stoller, 1978; Tester and Morris, 1987). When sunlight strikes the ground, the spectral characters of light are altered with depth under the soil surface (Figure 1A; Kasperbauer and Hunt, 1988; Mandoli et al., 1990). Photons in the red and far-red part of the spectrum can penetrate deeper than blue light photons. Furthermore, vascular tissue can conduct light to the roots over several centimeters and, again, red to far-red light reaches deeper than blue light (Briggs and Mandoli, 1984; Sun et al., 2003, 2005). Plants have evolved complex and extremely sensitive light sensing systems to react properly to light of different spectra. Plants have several classes of sensory photoreceptors, including the UV-B photoreceptor, UV-A/blue (B) light receptors and red (R)/far-red (FR) receptors (Briggs and Lin, 2012). Most members of these photoreceptors can be expressed in plant roots, giving roots the ability to sense light at wavelengths from the spectral UV-B to FR regions. For laboratory maintained *Arabidopsis* seedlings, when shoots and cotyledons are exposed to light and roots are grown in shadowed conditions, the root growth and the root-shoot ratio change prominently (Xu et al., 2013; Yokawa et al., 2013, 2014; Novák et al., 2015). Young seedlings with illuminated roots have shorter hypocotyls and longer roots (Novák et al., 2015). The shading roots condition was applied via new method “GLO-Roots” to analyze the root system architecture, showing that light changes the root architecture (Rellán-Álvarez et al., 2015). Importantly, the phot1 mutant is not affected by light exposure (Rellán-Álvarez et al., 2015). *Arabidopsis* roots exposed to continuous light generate immediate burst of reactive oxygen species (ROS) and show significantly altered responses to salt stresses (Yokawa et al., 2011, 2014). Moreover, Gundel et al. (2014) have hypothesized that the light perceived by the shoots and canopy cover could affect the root architecture. Glucose, synthesized by the photosynthetic process, influences root growth direction and root architecture by adjusting transport and response of phytohormones, for instance, brassinosteroids, auxin, cytokinin, and ethylene (Kircher and Schopfer, 2012; Roycewicz and Malamy, 2012; Singh et al., 2014a,b). Light, directly or/and indirectly, affects root growth, lateral root initiation, root hair formation and root gravitropic and phototropic bending (Lake and Slack, 1961; Klemmer and Schneider, 1979; Burbach et al., 2012; Hopkins and Kiss, 2012; Wan et al., 2012). Another newly proposed system for cultivating young *Arabidopsis* seedlings with shaded roots is a D–root system (Silva-Navas et al., 2015). In the D-Root system, the light comes from the top and shoots perceive the same amount and intensity of light whereas roots do not get any light. Only in the modified D-Root system, used to analyze specific wavelengths, the light is provided frontally (Silva-Navas et al., 2015).

Plants and their roots actively sense light and analyze both the spectrum and intensity of light using several photoreceptors, to integrate the development of organs growing aboveground and underground. In this review, we will discuss the spatial distribution of these photoreceptors and the physiological responses of roots to different light signals. It complements the more general reviews published recently (Kutschera and Briggs, 2012; Goyal et al., 2013; Briggs, 2014; Liscum et al., 2014; Fankhauser and Christie, 2015).

## Phytochromes

The plant sensory photoreceptor, phytochrome, was first discovered in the 1960s (Siegelman and Hendricks, 1964). Five members of this red/far-red photoreceptor family, phyA-phyE, are encoded by the nuclear genome in *Arabidopsis thaliana* (Briggs and Olney, 2001). All phytochromes use a single chromophore, phytochromobilin, to sense light signals (Lamparter, 2004). Phytochromes have two spectrally distinct conformations. The Pr form has a maximum absorption wavelength in the red spectrum (max = 660 nm), while Pfr absorbs far-red light (max = 730 nm) and these two conformations are photoconvertible. For example, quantification of seed germination rate under special light conditions revealed that Pfr is the active form initiating the germination of plants (Shinomura et al., 1994).

Schwarz and Schneider (1987) reported that phytochromes accumulated in the coleoptile tip, shoot apex and the root cap of *Zea mays*, while other root regions almost lack expression of phytochromes. Seven years later, expression of reporter genes driven by endogenous promoters was reported for phytochromes in different model plants. Adam et al. (1994) reported that the *PHYA* gene was mainly expressed in the root meristem and root cap in both light- and dark-grown *Nicotiana tabacum* seedlings. Later, Somers and Quail (1995a,b) agreed with this expression pattern of *PHYA* in *A. thaliana*, with additional *PHYA* expression in initiation sites of lateral roots. They further found that the expression of *PHYB* genes in meristem and root cap was induced by light illumination (Somers and Quail, 1995a,b). In addition, light stimulation of the dark-grown *Arabidopsis* seedlings induced the *PHYD* expression in whole roots with high expression rate in the root elongation and transition zones, but not in the root apical meristem and root cap (Goosey et al., 1997).

Phytochromes mediate variable physiological processes in roots of *Arabidopsis* seedlings, including promotion of root elongation, formation of lateral roots and mediation of root phototropic responses (Takano et al., 2001; Kiss et al., 2003a; Costigan et al., 2011; Raya-Gonzalez et al., 2014). PhyA and phyB act as active photoreceptors leading to red light-induced positive root phototropism (Kiss et al., 2003a). PhyA inhibits the blue light-induced negative root phototropism in *Arabidopsis* (Kiss et al., 2003b). Later, Correll and Kiss (2005) reported that both phyA and phyB have roles in light-stimulated root elongation, which is related to the root gravitropism. Hopkins and Kiss (2012) used mutant lines lacking PHY chromophore in shoots (CAB3::pBVR) and roots (M0062/UASBVR) to determine that the root growth was directly affected by the light sensing in roots. The signaling systems mediated by phys provide a mechanism to balance and integrate the development of shoots and roots (Salisbury et al., 2007). Interestingly, light signals sensed by the root apices influence also the shoot gravitropic bending (Hopkins and Kiss, 2012).

## Cryptochromes

Cryptochromes (CRYs) were discovered as a blue light photoreceptor in plants in the 1990s. CRYs are nuclear flavoproteins, with homology to photolyases, which exist in almost all organisms (Thompson and Sancar, 2002; Chaves et al., 2006; Ikeda et al., 2011; Christie et al., 2015). The model plant *Arabidopsis* has two members, cry1 and cry2 (Christie et al., 2015), which mediate inhibition of hypocotyl elongation under blue light, floral initiation controlled by circadian rhythms and other blue light-induced processes (Li and Yang, 2007; Yu et al., 2010). Crys have similar structures with two functional domains, the N-terminal PHR (photolyase-homologous region) domain that binds the chromophore FAD (flavin adenine dinucleotide) and a CCE (CRY C-terminal extension) domain at the C-terminal (Yu et al., 2010). Blue light changes the protein conformation by altering phosphorylation status, adjusting the interaction with protein partners, such as CIB1 (CRYPTOCHROME-INTERACTING bHLH1), COP1 (CONSTITUTIVELY PHOTOMORPHOGENIC 1), and SPAs (SUPPRESSOR OF PHYA), to activate the cry signaling pathways (Yu et al., 2007, 2010). cry3, or CRY-dash, is another putative member of the cry family, but its physiological roles are still unclear (Brudler et al., 2003; Huang et al., 2006).

Expression of *CRY1* and *CRY2* were detected in *Arabidopsis* roots at the transcriptional and post-transcriptional levels. It appears that *CRYs* affect root elongation via indirect pathways. The perception site of blue light is within the shoot, affecting root elongation by inhibiting rootward auxin transport (Canamero et al., 2006; Mao et al., 2014). However, more direct impacts of *CRYs* on root growth were also reported (Zeng et al., 2010a,b). Both cry1 and cry2 inhibit root growth, lower levels of free auxin and PIN1 amount, and increase flavonoids (Zeng et al., 2010b). Over-expression of *CRY1* and *CRY2* inhibit root growth and make it less sensitive toward auxin transport inhibitor NPA (Zeng et al., 2010a). In addition, cry1 restrains lateral root formation via inhibiting polar auxin transport (Zeng et al., 2010b). Interestingly, the blue light induced development of chloroplasts in root is synergistically mediated by crys and phys (Usami et al., 2004). Light signals are transduced via auxin/cytokinin signaling pathways to modify transcriptional factors LONG HYPOCOTYL5 (HY5) and GOLDEN2-LIKE2 (GLK2), initiating root greening processes (Kobayashi et al., 2012).

## Phototropins

Phototropins are blue light photoreceptors mediating dynamic plant behaviors, including shoot and root phototropism, chloroplast relocalization, adjustment of stomatal opening and expansion of cotyledons (Christie, 2007). The phototropin family has two members, phot1 and phot2. Both phots have similar structures and molecular mechanisms that sense blue light. They have two N-terminal LOV (light, oxygen, voltage) domains, which bind to flavin mononucleotide (FMN; Christie et al., 1999). The LOV domains are activated under illumination by forming a covalent bond between the cysteine residue and FMNs (Harper et al., 2003). The C-terminal kinase activities are released by activation of the LOV2 domain, causing self-phosphorylation or/and cross-phosphorylation of phototropins (Kaiserli et al., 2009). In addition to phototropin itself, several substrates of phot1-kinase were published in recent years. Among them, the ATP-BINDING CASSETTE B19 (ABCB19) is an auxin efflux transporter that can adjust the phototropic responses by maintaining the polar auxin transport in hypocotyls (Christie et al., 2011). The PHYTOCHROME KINASE SUBSTRATE 4 (PKS4) may have roles in adjusting phototropin- and phytochrome-mediated responses (Demarsy et al., 2012). However, PKS4 has only limited roles in the phototropic signaling process. BLUE LIGHT SIGNALING1 (BLUS1) is another known phosphorylation substrate of phots, mediating blue light-induced stomata opening of *Arabidopsis* (Takemiya et al., 2013). Besides these signaling proteins, phots need variable interaction proteins to mediate blue light signaling, including the NPH3 protein family (NPH3/RPT2/CPT1; Motchoulski and Liscum, 1999; Inada et al., 2004; Haga et al., 2005; Pedmale and Liscum, 2007), 14-3-3 proteins and small G-protein ARF proteins (Sullivan et al., 2009; Tseng et al., 2012). Tissue specific expression of phots provides another mechanism (see Figure 1C, for the root apex) to mediate a wide range of physiological processes (Sakamoto and Briggs, 2002; Wan et al., 2008; Preuten et al., 2013).

In mature *Arabidopsis* root system, phot1 is strongly expressed in the upper (closer to the soil surface) roots, where blue light reaches (Figure 1A), to increase drought tolerance in roots (Galen et al., 2007a,b). Moreover, mutant roots lacking phot1 showed random growth whereas control roots with active phot1 enjoyed directional and efficient growth (Galen et al., 2007a). In *Arabidopsis* root apices, phot1 accumulates in the apical region of primary and lateral roots, mediating blue light induced negative bending in primary roots and suppressing lateral root elongation (Sakamoto and Briggs, 2002; Wan et al., 2008; Zhang et al., 2013; Moni et al., 2015). Compared to the important roles of phot1 in roots, phot2 has only very weak distribution in root tissues (Kong et al., 2006; Wan et al., 2008). Interestingly, *Arabidopsis* root caps (Figures 1B,C) do not express phot1 (Sakamoto and Briggs, 2002; Wan et al., 2008), implying that the site of blue light perception is the root transition zone, adjusting root bending by controlling polar auxin transport (Figure 1: Wan et al., 2012; Zhang et al., 2013).

## Root Phototropism via Light-Activated Root Photoreceptors: Root Cap Versus Transition Zone

Both root and light tropisms have attracted the attention of researchers since a long time (Darwin, 1907; Kutschera and Briggs, 2012; Briggs, 2014). According to a classic study to quantify root phototropic behaviors in 166 plant species under unilateral white light illumination, 88 species showed no phototropic bending, while 72 had negative and 8 had positive phototropic bending responses (Schaefer, 1911). Naundorf (1940) reported that the root caps of sunflower seedlings are responsible for negative phototropic bending in roots. However, Schneider suggested that removing the apical 1 mm of the roots of maize plants did not result in altered phototropic bending behavior (Schneider, 1964). Mullen et al. (2002) designed a computer feedback system to maintain the root tip in a vertical position, and a fine optic fiber to control the exact light perception site of maize. They confirmed that the root cap is the organ of blue light perception in maize (Mullen et al., 2002).

**FIGURE 2:**
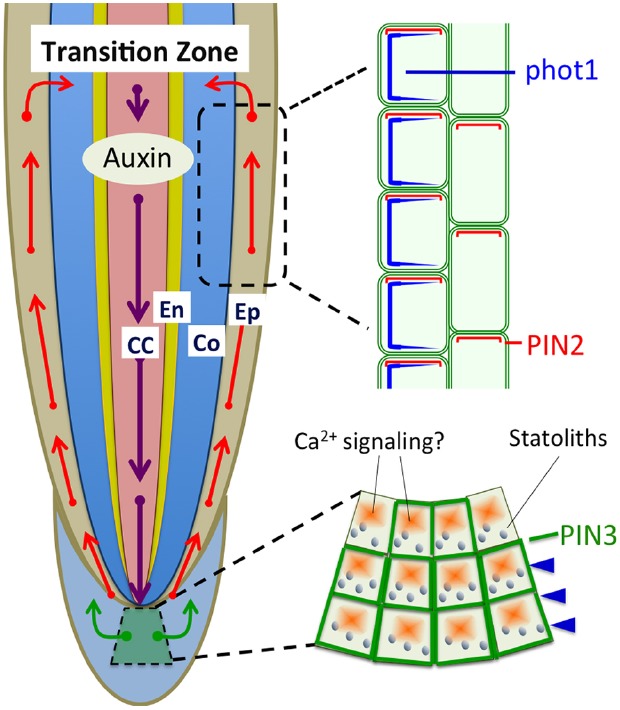
**Polar auxin transport based on PIN1, PIN2, and PIN3 is light sensitive and involved in the light-induced negative phototropism of roots (Friml et al., 2002; Wan et al., 2012; Zhang et al., 2014).** PIN1 is involved in the acropetal (rootward) auxin transport, PIN3 in the lateral auxin transport in statocytes, and PIN2 in the basipetal (shootward) auxin transport in epidermis and cortex cells. CC, central cylinder; En, endodermis; Co, cortex; Ep, epidermis.

Interestingly, the phot1-GFP protein driven by an endogenous promoter is not expressed in the root cap in *Arabidopsis*, but has a high expression level in the apical part of the transition zone (Figure 1C; Sakamoto and Briggs, 2002; Wan et al., 2008). The root cap, a site of perception of gravity signals (Swarup et al., 2005; Leitz et al., 2009), is rather a red-light-sensing organ as PHYA and PHYB are expressed there (Figures 1B,C and 2; Adam et al., 1994; Somers and Quail, 1995a,b; Goosey et al., 1997). Therefore, the critical question is this: is the root cap also a blue light-sensing organ? Is the root cap an organ for interaction between gravity and light signals to determine tropic bending?

The classic Cholodny–Went theory postulates that the asymmetric distribution of auxin determines both gravitropic and phototropic bending (Went and Thimann, 1937). It is logical to presume that phototropic and gravitropic signaling interacts via polar auxin transport in roots. The sensing of and response to tropic signals are spatially separated. In the maize root apex, the bending position responses to light and gravity signals have been determined, and gravitropic bending occurs at the transition zone (Baluška et al., 1996, 2010; Verbelen et al., 2006), also known as the distal elongation zone or the oscillatory zone (Baluška and Mancuso, 2013); whereas phototropic bending occurs above it, at the central elongation zone (Ishikawa and Evans, 1993; Mullen et al., 2002). The sensing of gravity occurs in the root caps. Re-orientation of roots causes sedimentation statoliths in the cortical endoplasmic reticulum to reorient to the new bottom in central S2 columella cells (Leitz et al., 2009). The dynamic force triggers release of Ca^2+^ from the endoplasmic reticulum, resulting in redistribution of PIN3 protein, an auxin efflux carrier, in these columella cells (Friml et al., 2002). However, the critical photoreceptor for blue light-induced root phototropism, phot1, is not expressed in root cap cells. Lateral blue light affects PIN3 localization in root cap columella cells, while the *phot1* mutant lacks this response (Zhang et al., 2013). How the phot1 sensing of blue light adjusts localization of PIN3 in columella cells is still unclear.

Furthermore, Wan et al. (2012) reported that activated phot1 determines the cellular behavior of PIN2 via the NPH3-based signal transduction process, affecting polar auxin transport in cortical cells of the root apex transition zone. Interestingly, PIN2 and phot1 are expressed preferentially in the transition zone (Figure 2), special root apex zone in which sensory-response integration is accomplished for root gravitropism and phototropism (Baluška et al., 2001, 2010; Wan et al., 2012; Baluška and Mancuso, 2013). Phot1-mediated signaling determines polar localization of PIN2 and PIN3, resulting in asymmetric auxin distribution on the shaded and lighted sides, leading to negative root phototropism. When roots were illuminated by symmetric blue light illumination, root gravitropic bending was reduced (Wan et al., 2012). Adjustment of the polar localization of PINs is a crucial process in polar auxin transport and PINOID (PID) kinase is one of the key regulators (Kleine-Vehn et al., 2009; Sukumar et al., 2009). Phot1 and PID belong to the same AGC kinase group, with a close evolutionary relationship (Galván-Ampudia and Offringa, 2007; Robert and Offringa, 2008). PID acts as a negative regulator of root apex phototropism and is expressed in the transition zone (Haga and Sakai, 2015). Intriguingly, blue light irradiation changed the symmetric PID distribution into an asymmetric one, with reduced PID on the shaded root apex side (Haga and Sakai, 2015). Thus, these results suggest that phototropic and gravitropic signaling may share a similar regulatory mechanism via a PIN-based auxin transport through the root apical zones.

Recently, PIN1 has also been shown to be needed for the root negative phototropism (Zhang et al., 2014). Blue light induced redistribution of both PIN2 (Wan et al., 2012), and PIN1 (Zhang et al., 2014) is mediated via BFA and GNOM-dependent endosomal trafficking pathways. Moreover, PINOID and PP2A are involved in the blue light induced redistribution of PIN1. Since PINOID is not expressed in stele cells expressing PIN1 (Dhonukshe et al., 2010; Zhang et al., 2014), it remains a mystery how PINOID controls the status of PIN1 protein.

Phys, the red light receptors, affect tropic bending via different mechanisms. Lariguet and Fankhauser (2004) revealed that top blue light illumination inhibited hypocotyl gravitropism through phyA-mediated pathways, while phot1 rescued upward growth by promoting phototropism. PhyA and phyB were found to have roles in adjusting root elongation and root gravitropism (Correll and Kiss, 2005). The phytochrome interaction protein, PKS1 acts as a signal transducer to inhibit gravitropic bending and adjust blue light-induced phototropism in *Arabidopsis* roots (Boccalandro et al., 2007). PhyA may mediate blue light responses by forming a signal complex with phot1 (Jaedicke et al., 2012). Interestingly, PKS1 is an interaction partner with phot1 and PKS4 is the substrate of phot1 kinase (Lariguet et al., 2006; Demarsy et al., 2012). Current structure analysis of the signaling protein complex may reveal the molecular mechanisms of phots, phys and PKSs interactions to reveal the mechanism of root phototropism and gravitropism.

## Light Spectra and Root Apex Functional Zones

Sunlight can penetrate the soil for centimeters. The spectrum and intensity of light underneath the soil surface can be sensed by roots, with diverse photoreceptors distributed specifically in different root zones. In addition to the phytochromes, CRYs and phototropin photoreceptors, studies showed that UV-B light affects root growth and development. Root development of UV-B sensitive1 (*rus1*, *rus2*) mutants of *Arabidopsis* is blocked under weak UV-B illumination (Tong et al., 2008; Leasure et al., 2009). The RUS1 protein is an essential factor for polar auxin transport in *Arabidopsis* (Yu et al., 2013). A UV-B receptor, UVR8, was identified and its crystal structure characterized (Christie et al., 2012). UVR8 is expressed in root apices of *Arabidopsis* (Rizzini et al., 2011; Yokawa et al., 2014) and its over-expression reduces growth of illuminated roots (Fasano et al., 2014).

Another protein, the F-box protein ZEITLUPE, uses the LOV domain to sense light, modulating circadian rhythms and mediating hypocotyl elongation under light conditions (Kim et al., 2007). However, we have little knowledge of these photoreceptors and their sensing mechanisms, especially in roots. The root apical region may act as a site to sense and respond properly to light signals of different intensities and wavelengths. Root caps express both phyA and phyB, allowing them to act as a sensing organ for red and blue light (Figure 1B). The root apex transition zone acts as a command center for interactions between sensory and endogenous signals (Baluška et al., 2010; Baluška and Mancuso, 2013). Polar localization of phot1 in the transition zone (Figure 1C) provides a fine adjustment mechanism for auxin polar transport in the root apex, influencing phototropism and gravitropism at the root apex. Since blue light cannot penetrate deep beneath the soil surface (Figure 1A), blue and red light receptors are more highly expressed in the upper portion of roots, adjusting formation and initiation of lateral roots to better cope with drought stress, and initiation development of chloroplast in roots.

In conclusion, plant roots can sense light and respond to a colorful underground world via complex signaling networks constructed from interwoven signaling pathways based on plant-specific photoreceptors. It is important to maintain the roots of laboratory-grown *Arabidopsis* seedlings in darkened Petri dishes (Yokawa et al., 2011, 2014; Xu et al., 2013: Novák et al., 2015). Illumination of roots affects not only roots but changes the whole seedlings, their metabolism, physiology and perhaps also their circadian rhythms.

### Conflict of Interest Statement

The authors declare that the research was conducted in the absence of any commercial or financial relationships that could be construed as a potential conflict of interest.
