# Corrigendum: How and why do root apices sense light under the soil surface?

**DOI:** 10.3389/fpls.2015.00930

**Published:** 2015-10-27

**Authors:** Mei Mo, Ken Yokawa, Yinglang Wan, František Baluška

**Affiliations:** ^1^College of Biological Sciences and Biotechnology, Beijing Forestry UniversityBeijing, China; ^2^Institute of Cellular and Molecular Botany, University of BonnBonn, Germany; ^3^Department of Biological Sciences, Tokyo Metropolitan UniversityTokyo, Japan

**Keywords:** root, photomorphogenesis, photoreceptors, plant, phytohormones, phototropism, auxin

Original sentence in Page 2, Paragraph 1, last sentence: However, these authors located their light source too close to the roots and also; importantly, not from the top (the shoot part) but rather from the side of roots which induced negative phototropism of roots, inhibiting the root growth. Therefore, the illuminated roots are shorter as the dark-grown roots in the D-root system (Silva-Navas et al., 2015).

Corrigendum:

In the D-Root system, the light comes from the top and shoots perceive the same amount and intensity of light whereas roots do not get any light. Only in the modified D-Root system, used to analyze specific wavelengths, the light is provided frontally (Silva-Navas et al., 2015).

The original article has been updated.

## Conflict of interest statement

The authors declare that the research was conducted in the absence of any commercial or financial relationships that could be construed as a potential conflict of interest.
